# Predictive Value of Odor Identification for Incident Dementia: The Shanghai Aging Study

**DOI:** 10.3389/fnagi.2020.00266

**Published:** 2020-08-26

**Authors:** Ding Ding, Zhenxu Xiao, Xiaoniu Liang, Wanqing Wu, Qianhua Zhao, Yang Cao

**Affiliations:** ^1^Institute of Neurology, Huashan Hospital, Fudan University, Shanghai, China; ^2^National Clinical Research Center for Aging and Medicine, Huashan Hospital, Fudan University, Shanghai, China; ^3^Clinical Epidemiology and Biostatistics, School of Medical Sciences, Örebro University, Örebro, Sweden

**Keywords:** olfactory, odor, dementia, prediction, logistic model, permutation importance method

## Abstract

**Objective:**

This study aimed to evaluate the value of odors in the olfactory identification (OI) test and other known risk factors for predicting incident dementia in the prospective Shanghai Aging Study.

**Methods:**

At baseline, OI was assessed using the Sniffin’ Sticks Screening Test 12, which contains 12 different odors. Cognition assessment and consensus diagnosis were conducted at both baseline and follow-up to identify incident dementia. Four different multivariable logistic regression (MLR) models were used for predicting incident dementia. In the no-odor model, only demographics, lifestyle, and medical history variables were included. In the single-odor model, we further added one single odor to the first model. In the full model, all 12 odors were included. In the stepwise model, the variables were selected using a bidirectional stepwise selection method. The predictive abilities of these models were evaluated by the area under the receiver operating characteristic curve (AUC). The permutation importance method was used to evaluate the relative importance of different odors and other known risk factors.

**Results:**

Seventy-five (8%) incident dementia cases were diagnosed during 4.9 years of follow-up among 947 participants. The full and the stepwise MLR model (AUC = 0.916 and 0.914, respectively) have better predictive abilities compared with those of the no- or single-odor models. The five most important variables are Mini-Mental State Examination (MMSE) score, age, peppermint detection, coronary artery disease, and height in the full model, and MMSE, age, peppermint detection, stroke, and education in the stepwise model. The combination of only the top five variables in the stepwise model (AUC = 0.901 and sensitivity = 0.880) has as a good a predictive ability as other models.

**Conclusion:**

The ability to smell peppermint might be one of the useful indicators for predicting dementia. Combining peppermint detection with MMSE, age, education, and history of stroke may have sensitive and robust predictive value for dementia in older adults.

## Introduction

Olfactory dysfunction is a common feature of neurodegenerative diseases, especially in dementia (e.g., Alzheimer’s disease, dementia with Lewy bodies, and Parkinson’s disease dementia), and is considered to be a premotor sign of neurodegeneration (Attems et al., 2014). Previous hospital- and population-based studies have demonstrated the association of olfactory dysfunction with dementia, cognitive decline, or mild cognitive impairment (MCI). Some human studies show a relationship between peppermint aroma stimulation and enhanced memory and functional performance in older people with dementia (Herz, 1997; Collier, 2007; Moss et al., 2008). Furthermore, peppermint’s preservation of central nervous system microglia as a mediator of improved cognitive function has also been reported by an experimental *in vivo* study (Koo et al., 2001). At the baseline of our Shanghai Aging Study, we found a lower score on the olfactory identification (OI) test and a reduced ability to identify odors of peppermint, orange, pineapple, cinnamon, coffee, fish, banana, rose, leather, and licorice in participants with MCI compared to those with normal cognition (Liang et al., 2016). We further verified these findings in the 5-year prospective phase and explored the association of inability to smell peppermint with a higher dementia onset risk (HR = 2.67, 95% CI: 1.44, 4.96) by using a multivariable logistic regression (MLR) model (Liang et al., 2020). However, the previous study also did not evaluate the performance (or predictive value) of peppermint in predicting incident dementia.

Variable selection is one of the core concepts in statistical learning, and it impacts the performance of predictive models significantly. Irrelevant or partially relevant variables may reduce the predictive ability of the models. There are many variable-selection methods available in data science, such as recursive feature elimination, principle component analysis, correlation matrix with heat map, feature importance, and some wrapper methods (Hua et al., 2009; Liu and Motoda, 2012). Variable importance is straightforward and can be easily explained to an audience outside of the fields of data science and informatics. In the current study, the permutation importance (PI) method, which permutes the values of a feature of interest and reevaluates the predictive ability of the models (Altmann et al., 2010), was used to evaluate the importance of the OI test, certain odors, and other known risk factors for predicting incident dementia in the prospective Shanghai Aging Study.

## Materials and Methods

### Study Setting and Participants

The Shanghai Aging Study is a prospective cohort study aiming to enumerate the prevalence, incidence, and risk factors for dementia and MCI among residents aged ≥60 in an urban community of central Shanghai. The study design and participant recruitment of SAS are described in detail elsewhere (Ding et al., 2014; Liang et al., 2016; Liang et al., 2020). A flowchart of recruitment for study participants is shown in Supplementary Figure S1. In total, 1,782 recruited participants without dementia completed both cognitive assessment and the OI test at baseline (2010–2011). The participants were contacted between April 1, 2014 and December 31, 2016, to investigate the first wave of dementia incidence. After excluding participants who were lost to follow-up, deceased, or had missing values in the analysis variables, 947 participants were included in the current study. After an average of 4.9 years of follow-up, 75 (7.0%) of the 947 included participants were diagnosed with new-onset dementia with an incidence rate of 16 [95% confidence interval (CI): 13–20] per 1,000 person-years. Participants with incident dementia were older (mean age: 77.8 ± 5.6) than participants without incident dementia (mean age: 69.9 ± 6.5, *p* < 0.001) at the baseline.

### Collection of Baseline Data

*Demographics and lifestyle:* Demographic and lifestyle characteristics, including age, sex, years of formal education, cigarette smoking, and alcohol consumption, were collected via an interviewer-administered questionnaire (Shu et al., 2004).

*Physical measurements:* Each participant’s height and weight at baseline were measured by a research nurse. BMI was calculated as weight in kilograms divided by height in meters squared.

*Medical history:* Participants’ medical histories, including physician-diagnosed hypertension, coronary artery disease (CAD), diabetes, and stroke were asked by neurologists from the Department of Neurology, Huashan Hospital (Liang et al., 2020).

*Apolipoprotein (APOE) genotype:* DNA was extracted from blood or saliva samples at baseline. APOE genotyping was conducted by the Taqman SNP method (Smirnov et al., 2009). The presence of at least one *ε*4 allele was defined as APOE-*ε*4 allele positive.

*OI test:* OI at baseline was assessed using the Sniffin’ Sticks Screening Test 12 (SSST-12), which consists of 12 odors (orange, leather, cinnamon, peppermint, banana, lemon, licorice, coffee, cloves, pineapple, rose, and fish) presenting on felt-tip sticks (Wolfensberger, 2000). The SSST-12 kit was purchased from Burghart Medical Technology, Hamburg, Germany (Tinsdaler Weg 175, 2020). OI was defined as an individual correctly naming an odor or odors, either with or without the help of alternative choices. The administration of SSST-12 is described in detail elsewhere (Liang et al., 2016).

*Cognition assessment and consensus diagnosis:* The cognitive function of the participants was assessed using a battery of neuropsychological tests, including the Mini Mental State Examination (MMSE) (Tombaugh and McIntyre, 1992), Conflicting Instructions Task (go/no-go task), Stick Test, Modified Common Objects Sorting Test, Auditory Verbal Learning Test, Modified Fuld Object Memory Evaluation, Trail-Making Test A & B, and Renminbi (Chinese Currency) Test. The normative data and detailed description of the assessment battery are reported elsewhere (Zhang et al., 1990; Ding et al., 2015). Each participant’s mood was evaluated using the Zung Self-Rating Anxiety Scale and the Center for Epidemiologic Studies Depression Scale (CESD), and depression was present if a CESD score ≥16 (Zung, 1971; Eaton et al., 2004).

Two study neurologists, one neuropsychologist, and one neuroepidemiologist reviewed the functional, medical, neurological, psychiatric, and neuropsychological data of the participants and reached a consensus regarding the presence of dementia using the Diagnostic and Statistical Manual of Mental Disorders IV (DSM–IV) criteria (American Psychiatric Association, 1994).

### Prospective Follow-Up

Between April 2014 and December 2016, participants who were diagnosed as dementia-free were invited for a clinical interview as the first wave of follow-up to detect incident dementia cases. Each participant was administered the same neuropsychological battery for the cognition assessment. Procedure and criteria of the consensus diagnosis were identical with that at baseline.

### Statistical Analysis

#### Descriptive Analysis

Participants’ demographics, lifestyle, medical history, and OI test results are presented using mean with standard deviation (SD) or median with interquartile range (IQR) for the continuous variable and using a percentage for the categorical variables. Difference between groups was tested using the chi-squared test for categorical variables and analysis of variance (ANOVA) or Mann–Whitney *U* test for continuous variables. Correlation was measured using the Pearson correlation coefficient between two continuous variables and using the point-biserial correlation coefficient between a binary and a continuous variable (Demirtas and Hedeker, 2016) and the phi coefficient between two binary variables (Chen and Popovich, 2002). Multicollinearity between the variables is presented using a heat map. A two-sided *P*-value <0.05 is considered statistically significant.

#### Determination of Variable Importance

*Prediction:* In the current study, prediction for dementia incidence was conducted using MLR analysis. Four types of MLR models were constructed in the study. In the first or no-odor model, we only included demographics, lifestyle, and medical history variables (i.e., sex, age, BMI, height, education, smoking, drinking, CAD, hypertension, diabetes, depression, stroke, APOE-ε4, and MMSE) but not any odor. In the second type or single-odor model, we added only one single odor or OI sum score to the first model. In the third or full model, all 12 odors were included. Weight was excluded in the first three types of models, and OI sum score was excluded in the first and third models because of high collinearity with other variables. In the fourth model, the variables were selected using a bidirectional stepwise selection method (Zhang, 2016).

*Validation:* The K-fold cross-validation method was used during the MLR model learning and validation, which is a standard way to obtain unbiased estimates of a model’s goodness of fit and to handle the overfitting problem in statistical learning. In brief, we randomly split the data set into five equal partitions and constructed an MLR model on four partitions while validating it on the remaining partition. In each iteration, the prediction was made for the one held-out partition. In the end, we got the prediction for the whole data set and used it for validation (James et al., 2013).

*Evaluation:* The metrics, including sensitivity, specificity, accuracy, and area under the receiver operating characteristic (ROC) curve, were used to evaluate the models’ predictive ability. Terminology and derivations of the metrics are given in detail elsewhere (Cao et al., 2019). The acceptable, good, and great prediction models for incident dementia are defined as the area under the ROC curve (AUC) of a model greater than 0.7, 0.8, and 0.9, respectively (Marzban, 2004; Mandrekar, 2010).

*Variable standardization:* Because scalability is an important aspect of statistical learning and matters for the models’ performance, variable standardization is preferred before training the models (Lantz, 2013). Because the aim of the current study was to evaluate the predictive ability of the models rather than to interpret the associations between the predictors and the outcome, therefore, all features were treated as continuous or discrete numerical variables and were scaled using the standard scaler to have a mean of 0 and a SD of 1 (Zheng and Casari, 2018).

*PI:* For the MLR models, PI was calculated for each variable, which is measured by looking at how much the accuracy decreases when the information on the variable is not available (Altmann et al., 2010). To mask the information on a variable during validation, instead of removing the variable from the data set, the PI method replaces it with random noise by shuffling the values of the variable, i.e., using values from other participants (Breiman, 2001; Fisher et al., 2019). The relative importance of a variable was calculated as the accuracy decrease of the variable relative to the range of the accuracy decreases of all the variables (Gómez-Ramírez et al., 2019).

#### Software and Hardware

The descriptive analyses were performed using Stata 16.0 (StataCorp LLC, College Station, TX, United States). The MLR models and PI evaluation were achieved in Python 3.6 (Python Software Foundation^1^) using packages scikit-learn 0.22.1 (Pedregosa et al., 2011) and ELI5 0.10.1 (Korobov and Lopuhin, 2020). All computation was conducted on a computer with a 64-bit Windows 7 Enterprise operating system (Service Pack 1), Intel^®^ Core TM i5-4210U CPU of 2.40 GHz, and 16.0 GB installed random access memory.

### Ethical Consideration and Data Availability

The study is an observational study and was approved by the Medical Ethical Committee of Huashan Hospital, Fudan University, Shanghai, China (approval number: 2009-195). All participants and/or their legal guardian gave their written informed consent for participation in the study. There is no personal identification disclosed in our data. The data are not publicly available but may be available upon reasonable request and with permission of the Ding Ding (dingding@huashan.org.cn).

## Results

### Characteristics of the Participants

**FIGURE 1:**
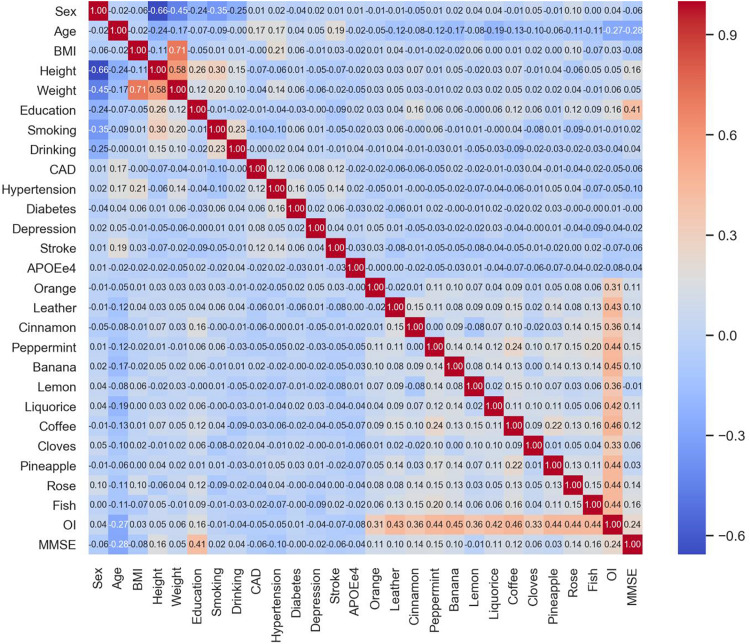
Heat map of the pairwise correlation coefficients between the variables.

Detailed baseline information on the participants is published elsewhere and given in Supplementary Table S1 (Liang et al., 2020). In general, compared to those who did develop dementia (*n* = 872), participants with incident dementia (*n* = 75) were older (77.8 vs 69.9 years), shorter (156.5 vs 162.0 cm), weighed less (59.1 vs 64.4 kg), and had less education (9 vs 12 years) when recruited. CAD, stroke, and APOE-*ε*4 positive were more frequently observed in the new-onset dementia cases (Supplementary Table S1). The new-onset dementia cases had a lower correct identification rate for most odors and lower OI sum and MMSE scores at the baseline (Supplementary Table S1). There was no significant multicollinearity observed between the variables except for the high correlation between height and weight, weight and BMI, and OI sum score and the 12 odors (Figure 1).

### Predictive Ability of the Models

The regression coefficients of the full and stepwise models are shown in Tables 1 and 2. In the full model, age, APOE-*ε*4, peppermint, pineapple, banana, and MMSE are statistically significantly (at the two-sided type one error α = 0.05 level) associated with dementia incidence. However, in the analysis, wrong identification of pineapple is associated with a lower probability of dementia incidence (Table 1).

**TABLE 1:** Multivariable logistic regression coefficients of the full model.

Variable	β	SE	*P*-value
Sex	–0.051	0.231	0.824
Age	1.086	0.200	0.000
BMI	–0.240	0.162	0.139
Height	–0.361	0.211	0.086
Education	–0.241	0.146	0.099
Smoking	0.331	0.185	0.074
Drinking	–0.288	0.213	0.176
CAD	0.099	0.123	0.424
Hypertension	–0.068	0.166	0.683
Diabetes	–0.098	0.160	0.539
Depression	0.244	0.138	0.076
Stroke	0.201	0.128	0.116
APOE-*ε*4	0.352	0.139	0.011
Orange	0.055	0.151	0.715
Leather	–0.235	0.163	0.151
Cinnamon	–0.182	0.179	0.308
Peppermint	–0.371	0.118	0.002
Banana	–0.341	0.153	0.026
Lemon	0.202	0.168	0.229
Licorice	0.054	0.160	0.737
Coffee	–0.027	0.127	0.828
Cloves	–0.013	0.161	0.934
Pineapple	0.408	0.176	0.020
Rose	–0.244	0.158	0.124
Fish	0.159	0.148	0.280
MMSE	–0.719	0.138	0.000

*SE, standard error; BMI, body mass index; CAD, coronary artery disease; OI, olfactory identification sum score; APOE, apolipoprotein; MMSE, Mini-Mental State Examination. Weight and olfactory identification sum score were excluded from the model because of collinearity. β corresponds to the change of women versus men for sex; per year for age and education; per unit for BMI, height, and MMSE; with the feature versus without the feature for smoking, drinking, and comorbidities; and positive versus negative for APOE-*ε*4 and individual odor tests.*

**TABLE 2:** Multivariable logistic regression coefficients of the stepwise model.

Variable	β	SE	*P*-value
Age	1.057	0.186	0.000
Weight	–0.360	0.171	0.036
Education	–0.308	0.132	0.020
Depression	0.248	0.133	0.061
Stroke	0.209	0.121	0.086
APOEe4	0.311	0.137	0.023
Leather	–0.255	0.157	0.104
Peppermint	–0.331	0.111	0.003
Banana	–0.310	0.148	0.036
Lemon	0.261	0.158	0.098
Pineapple	0.406	0.166	0.014
Rose	–0.241	0.149	0.107
MMSE	–0.693	0.133	0.000

*SE, standard error; APOE, apolipoprotein; MMSE, Mini-Mental State Examination. β corresponds to the change per year for age and education, per unit for weight and MMSE, with versus without depression or stroke, and positive versus negative for APOE-*ε*4 and individual odor tests.*

**FIGURE 2:**
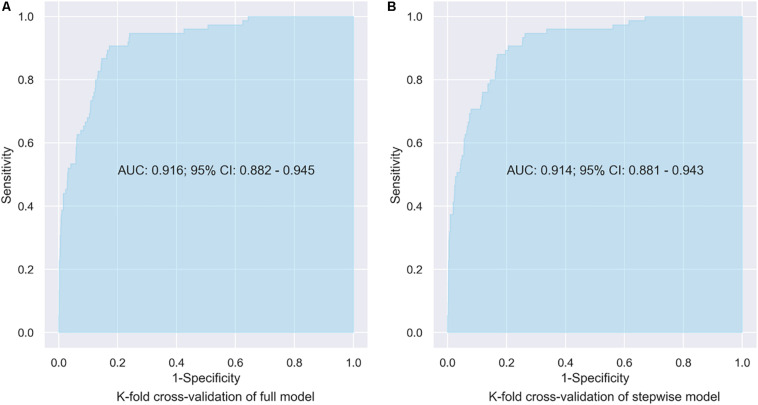
AUC of the full and stepwise models.

In the stepwise model, age, weight, education, APOE-*ε*4, peppermint, banana, pineapple, and MMSE are associated with dementia incidence. Similarly, wrong identification of pineapple is associated with lower probability of dementia incidence (Table 2). The predictive abilities of the four types of models are shown in Table 3. There is no significant difference in predictive abilities between the no-odor and the single-odor models; both types of models show great ability for predicting dementia incident (AUCs ranging between 0.901 and 0.906). However, the model including licorice shows higher accuracy (= 0.818), and the model including banana, lemon, or cloves shows higher sensitivity (= 0.920, Table 3). The predictive abilities of the full and stepwise models are similar (AUC = 0.916 and 0.914, respectively) (Figure 2) and better (although not significant) than those of the no- or single-odor models (Table 3).

**TABLE 3:** Performance matrix of the prediction models.

	Sensitivity	Specificity	Accuracy	AUC	95% CI of AUC	
					
					Lower limit	Upper limit
No-odor model^a^	0.893	0.791	0.799	0.901	0.864	0.933
**Single-odor model^b^**						
Orange	0.880	0.791	0.798	0.901	0.867	0.933
Leather	0.880	0.804	0.810	0.905	0.870	0.935
Cinnamon	0.893	0.804	0.811	0.904	0.867	0.936
Peppermint	0.893	0.781	0.790	0.904	0.866	0.936
Banana	0.920	0.768	0.780	0.906	0.873	0.936
Lemon	0.920	0.749	0.762	0.902	0.869	0.933
Licorice	0.867	0.814	0.818	0.902	0.869	0.932
Coffee	0.893	0.786	0.794	0.900	0.867	0.932
Cloves	0.920	0.749	0.762	0.902	0.866	0.932
Pineapple	0.893	0.796	0.804	0.903	0.866	0.932
Rose	0.893	0.786	0.794	0.904	0.869	0.936
Fish	0.893	0.791	0.799	0.902	0.864	0.933
OI	0.933	0.751	0.766	0.904	0.869	0.935
Full model^c^	0.907	0.828	0.834	0.916	0.882	0.945
Stepwise model^d^	0.880	0.831	0.835	0.914	0.881	0.943
Simple model^e^	0.880	0.781	0.790	0.901	0.859	0.931

*^*a*^Predictors: sex, age, body mass index (BMI), height, education, smoking, drinking, coronary artery disease (CAD), hypertension, diabetes, depression, stroke, apolipoprotein (APOE)-*ε*4, and Mini-mental State Examination (MMSE). ^*b*^Other predictors are the same as in the no-odor model. ^*c*^Predictors: sex, age, BMI, height, education, smoking, drinking, CAD, hypertension, diabetes, depression, stroke, APOE-*ε*4, MMSE, and the 12 odors. ^*d*^Predictors: age, weight, education, depression, stroke, APOE-*ε*4, leather, peppermint, banana, lemon, pineapple, rose, and MMSE. ^*e*^Predictors: MMSE, age, peppermint, stroke, and education.*

### Relative PI of the Variables

**FIGURE 3:**
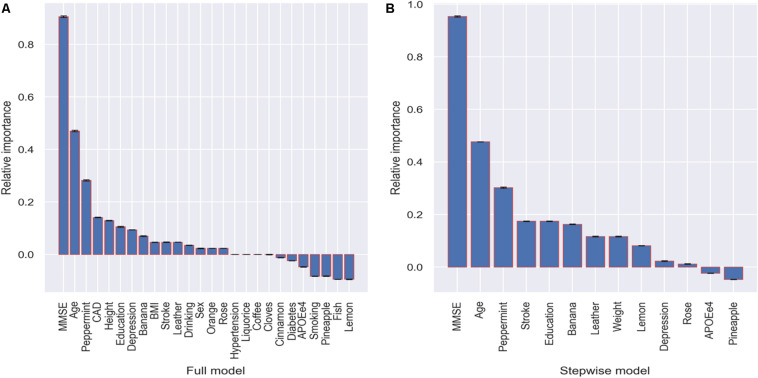
Relative importance with corresponding standard deviation of the variables in terms of accuracy decrease in prediction.

The relative importance of the variables was evaluated in the full and stepwise models because they showed the best performance for prediction. In the full model, the five most important variables are MMSE, age, peppermint, CAD, and height (Figure 3). In the stepwise model, the five most important are MMSE, age, peppermint, stroke, and education. Both results indicate that identification of peppermint odor might be an important indicator for dementia only after MMSE and age. In addition, banana also shows relative higher importance in both models (Figure 3). There are also variables with negative importance, which means that, when they were excluded from the model, the accuracy of the prediction increased.

When using a simple model that only includes the five most important variables in the stepwise model, we achieved a predictive ability (AUC = 0.901 and sensitivity = 0.880) as great as those of the aforementioned models that include many more variables (Table 3).

## Discussion

Although there is a study using random forest and permutation-based methods to select important variables for predicting conversion to MCI (Gómez-Ramírez et al., 2019), to the best of our knowledge, this is the first study investigating the predictive rather than associative value of the odors in the OI test for incident dementia in the elderly. There are several strengths in our study. First, the permuting destroys the interaction effects between variables, which means that it automatically takes into account both the main effect of a variable and the interaction effects with other variables on model performance (Fisher et al., 2019). Second, our data suggest that, not only is MMSE generally applicable to predict dementia in our geriatric population, but the addition of the ability to smell peppermint further improves the precision and accuracy of the model. This has tangible clinical benefits in both informing clinical decision making and translating statistical probability into coherent information for the elderly and their families. Thus, a consensus plan (either medical treatment or preventive intervention) may be more readily reached. Third, cross-validation was used when we evaluated the performance of the models, which minimized overfitting. Finally, different MLR models were constructed and compared, and they presented similar results, which ensure that our conclusion is conservative and robust.

### OI Test and Dementia Prediction

Previous studies reveal that both olfactory and certain cognitive functions are controlled via the orbitofrontal cortex, and reduced olfactory ability and manifestation of dementia are associated with brain changes in the hippocampus and entorhinal cortex (Rupp et al., 2006; Maurage et al., 2011; Seligman et al., 2013; Marigliano et al., 2014; Growdon et al., 2015). Olfactory dysfunction is associated with pathological features of cognitive impairment (Passali et al., 2015; Reijs et al., 2017). Some studies suggest that olfactory dysfunction could be a suitable biomarker for predicting cognitive impairment and development of dementia (Suzuki et al., 2004; Eibenstein et al., 2005; Devanand et al., 2015; Ottaviano et al., 2016; Roberts et al., 2016; Roalf et al., 2017). Our previous study also indicates that some odors, such as peppermint in the OI test, are associated with incident dementia in the older population (Liang et al., 2020). However, the predictive ability of the models incorporating the OI test was not ideal in previous studies. A large sample size (*N* = 2227) prospective study of middle-aged to older adults (55–86 years) concludes that olfactory function may serve as a marker for screening persons at high risk for cognitive decline and dementia. However, the AUC values in the study are only between 0.55 and 0.62 for the five cognitive tests (Tebrugge et al., 2018). In another prospective study of 757 participant aged 65 years and older, the University of Pennsylvania Smell Identification Test combined with the Selective Reminding Test–total immediate recall shows an improved performance for predicting dementia incidence; however, the AUC is still only 0.77 (Devanand et al., 2015). Although Stanciu et al. (2014) concludes that OI could independently predict conversion to dementia within a 10-year time span, the accuracy of the prediction is not evaluated in that study.

The SSST-12 test comprises 12 common and familiar odorants recognized by a majority of the population (Oleszkiewicz et al., 2019). The number of odors for selection could be as many as 37 in comprehensive olfactory tests (MediSense, 2020); however, it remains uncertain how many items are sufficient for a valid diagnosis or screening (Lotsch et al., 2016). Several studies attempt to reduce the number of odor identification items to 1–5 odors (Doty et al., 1996; Simmen et al., 1999; Hummel et al., 2001; Gilbert et al., 2002; Jackman and Doty, 2005; Mueller and Renner, 2006), and a recent study recommends a three-odor test with cinnamon as the best scoring odor (Lotsch et al., 2016). Although an inability to identify certain odors has previously been used as a predictor for incident dementia (Adams et al., 2018; Liang et al., 2020), the relative importance of the odors compared to each other or compared to other predictors has not been investigated.

### Peppermint and Dementia

The current study further confirms previous findings that the ability to smell peppermint may play an important role in predicting dementia incidence in the elderly (Adams et al., 2018; Liang et al., 2020). It also reveals that peppermint is the third most important variable in the prediction models, only after MMSE and age. Using a simplified prediction model including MMSE, age, peppermint, stroke, and education, the specificity of the prediction can be as high as 0.88 with an AUC of 0.90.

The relationship between detection of peppermint and dementia has been investigated previously. A human study of peppermint’s modulation on long-term potentiation shows a direct correlation between peppermint oils and enhanced memory (Moss et al., 2008). In recall tests of extended memory, improved cognitive function arises in response to exposure to peppermint aroma during both learning and memory retrieval tasks (Herz, 1997). In a randomized single blind trial, researchers used multisensory stimulation, including aromatic cloves or peppermint, to improve functional performance in older people with dementia, and they find a significant effect of the intervention on function, mood, and behavior in people with a diagnosis of moderate/severe dementia (Collier, 2007). However, Fox et al. (2012) study suggests that consumption of peppermint does not mediate alertness or enhance cognitive performance. Although the underlying mechanism of the effects of peppermint on neurological functions is not clear yet, an experimental study shows that *in vivo* exposure of glial cells to peppermint oil might inhibit heat shock-induced apoptosis of astrocytes in rat and human cell models, suggesting peppermint’s preservation of central nervous system microglia as a mediator of improved cognitive function (Koo et al., 2001). Further research investigating compound metabolism is required to optimize quantification of memory performance following peppermint ingestion.

### MLR and PI

There are other statistical learning methods for prediction, such as discriminant analysis, decision tree, K-nearest neighbor, support vector machine, and multilayer perceptron (James et al., 2013). The reasons for using MLR in the current study are that (a) logistic regression is the most widely used method in diagnostic tests and prediction studies for binary outcomes in medical science. The results from a logistic regression analysis can be easily comprehended by clinical researchers (Coughlin et al., 1992; Greiner et al., 2000; Janssens et al., 2005). (b) Coefficients from the logistic regression models can be translated into odds ratios, which are widely used in medical and epidemiology studies (Hilbe, 2009).

Compared to Gini importance, which is model-agnostic and embedded in tree-based statistical learning algorithms, such as random forest (Nembrini et al., 2018), the concept of PI is straightforward. PI measures the importance of a variable by calculating the decrease in the model’s prediction accuracy after permuting the variable. A variable is “important” if shuffling its values decreases the accuracy because, in this case, the model relies on the variable for the prediction (Breiman, 2001). Although permuting irrelevant or partially relevant variables may increase the predictive ability of the models, it may result in a negative importance, just as we observe in Figure 3. The method is generalizable no matter the predictive model and most suitable for computing variable importance when the number of variables is not large; otherwise, it can be resource-intensive (Altmann et al., 2010; Fisher et al., 2019).

Because using a limited number of variables may have already achieved great prediction for dementia incidence (such as the simple model in Table 3), one single variable contributes little to the improvement accuracy of the prediction in a multivariable model; therefore, we only compare relative importance of the variables in this study. It is useful when we want to find common important variables using different statistical learning methods. In our study, all five of the most important variables (MMSE, age, peppermint, stroke, and education) found in the stepwise MLR model are consistent with the statistically significant risk factors derived from previous studies (Snowdon and Nun, 2003; Cullen et al., 2005; Ngandu et al., 2007; Mijajlovic et al., 2017; Liang et al., 2020). The combination gives us real, predictive values that may be useful in clinical practice.

### Limitations

There are also several limitations in the study. First, the sample size is relatively small, and only 75 participants were diagnosed with new-onset dementia after an average of 4.9 years of follow-up. Essentially, the performance of statistical learning methods relies on the amount of data available. The more observations and variables, the better the models perform. Although we obtain satisfactory accuracy from the models, the generalizability of the findings is limited by the small sample size. Second, nominal variables are treated as discrete numerical features in this study. Although it increases the accuracy of prediction, the interpretability of the models is reduced. Third, about half of the participants who were lost to follow-up were excluded from our analysis data set. We do not know the incidence of dementia among the excluded participants and whether being lost to follow-up was associated with certain cognitive impairments. Although there is no statistically or clinically significant difference between the included and excluded participants in terms of demographic and lifestyle characteristics, the validity of the models is limited by the incompleteness and needs to be examined using data with better representativeness.

## Conclusion

The ability to smell certain odors, especially peppermint, might be one of the useful indicators for predicting dementia in the elderly. Incorporating peppermint with MMSE, age, education, and history of stroke, we may predict long-term dementia onset in older adults precisely. Aromatherapy using essential oils, including peppermint, to prevent and/or control symptoms of dementia deserves further investigation.

## Data Availability Statement

The data analyzed in this study is subject to the following licenses/restrictions: The data are not publicly available but may be available upon reasonable request and permission. Requests to access these datasets should be directed to DD, dingding@huashan.org.cn.

## Ethics Statement

The studies involving human participants were reviewed and approved by the Medical Ethical Committee of Huashan Hospital, Fudan University, Shanghai, China (approval number: 2009-195). The patients/participants provided their written informed consent to participate in this study.

## Author Contributions

YC and DD: conceptualization, methodology, and writing – original draft. DD and XL: data curation. YC: formal analysis and software. DD and QZ: funding acquisition. DD, XL, WW, and ZX: investigation. DD: project administration. YC, DD, and ZX: writing – review and editing. All authors contributed to the article and approved the submitted version.

## Conflict of Interest

The authors declare that the research was conducted in the absence of any commercial or financial relationships that could be construed as a potential conflict of interest.

## Footnotes

1 https://www.python.org/

## Supplementary Material

FIGURE S1 Flowchart of recruitment of participants.

TABLE S1 Baseline characteristics of participants with and without incident dementia.
