# Supplementary material for: Predictive Value of Odor Identification for Incident Dementia: The Shanghai Aging Study
Source: Front Aging Neurosci. 2020 Aug 26;12:266. doi: 10.3389/fnagi.2020.00266 (PMC7479092; doi:10.3389/fnagi.2020.00266)
Supplement: FIGURE S1 — Flowchart of recruitment of participants. [file Data_Sheet_1.PDF]

Suppl. Figure and Table

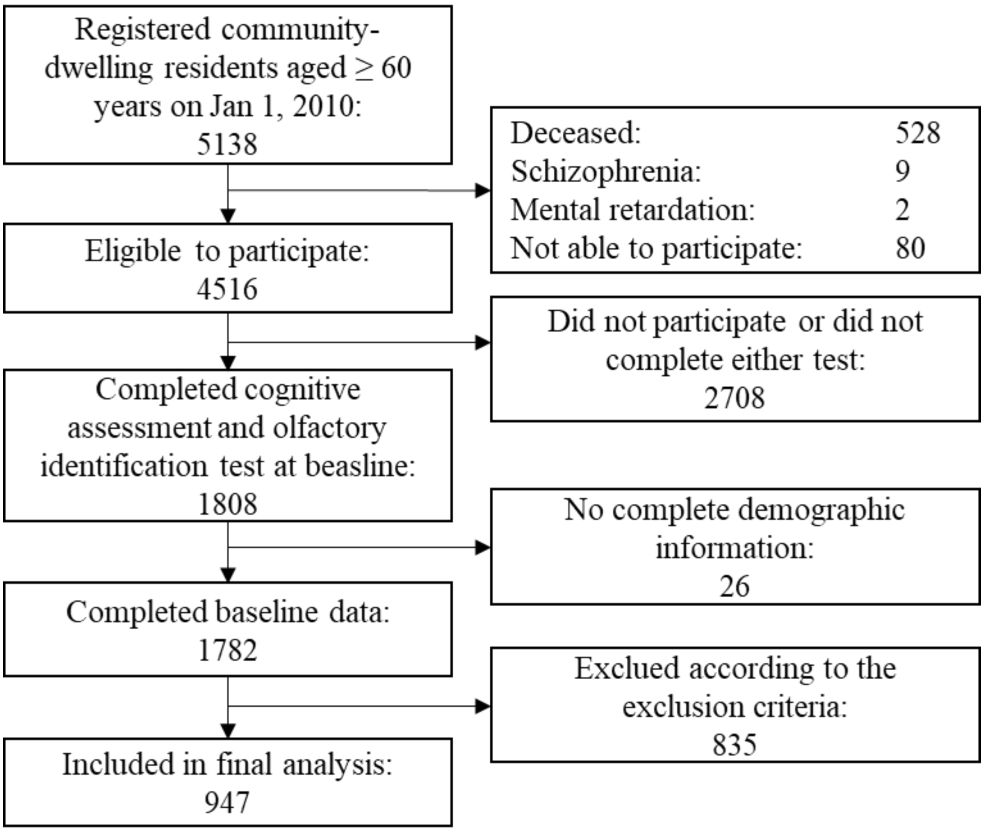

Figure S1. Flowchart of recruitment of participants

Table S1. Baseline characteristics of participants with and without incident dementia.

Variable	Participants without incident dementia (n=872)	Participants with incident dementia (n=75)	P-value
Male, n (%)	407 (46.7)	29 (38.7)	0.225
Age (year), mean (SD)	69.9 (6.5)	77.8 (5.6)	<0.001
BMI (kg/m^2), mean (SD)	24.49 (3.5)	24.48 (7.0)	0.988
Height (cm), mean (SD)	162.0 (8.4)	156.5 (13.2)	<0.001
Weight (kg), mean (SD)	64.4 (11.5)	59.1 (12.2)	<0.001
Education (year), median [IQR]	12.0 [12.0, 15.0]	9.0 [6.0, 12.5]	<0.001
Smoking, n (%)	93 (10.7)	8 (10.7)	1.000
Drinking, n (%)	83 (9.5)	4 (5.3)	0.319
CAD, n (%)	78 (8.9)	13 (17.3)	0.031
Hypertension, n (%)	451 (51.7)	48 (64.0)	0.054
Diabetes, n (%)	113 (13.0)	10 (13.3)	1.000
Depression, n (%)	120 (13.8)	16 (21.3)	0.105
Stroke, n (%)	92 (10.6)	19 (25.3)	<0.001
APOE-ε4positive, n (%)	137 (15.7)	19 (25.3)	0.046
Orange, n (%)	687 (78.8)	55 (73.3)	0.340
Leather, n (%)	504 (57.8)	30 (40.0)	0.004
Cinnamon, n (%)	397 (45.5)	20 (26.7)	0.002
Peppermint, n (%)	808 (92.7)	54 (72.0)	<0.001
Banana, n (%)	578 (66.3)	31 (41.3)	<0.001
Lemon, n (%)	468 (53.7)	40 (53.3)	1.000
Liquorice, n (%)	478 (54.8)	29 (38.7)	0.010
Coffee, n (%)	809 (92.8)	60 (80.0)	<0.001
Cloves, n (%)	465 (53.3)	32 (42.7)	0.098
Pineapple, n (%)	603 (69.2)	52 (69.3)	1.000
Rose, n (%)	570 (65.4)	33 (44.0)	<0.001
Fish, n (%)	717 (82.2)	53 (70.7)	0.021
OIS, median [IQR]	8.0 [7.0, 10.0]	7.0 [5.0, 8.0]	<0.001
MMSE, median [IQR]	29.0 [28.0, 30.0]	27.0 [25.0, 28.5]	<0.001

SD, standard deviation; BMI, body mass index, IQR, interquartile range; CAD, coronary artery disease; APOE, apolipoprotein; OIS, olfactory identification sum score; MMSE, Mini-mental State Examination score.
